# Nanogold Functionalized With Lipoamide-*iso*DGR: A Simple, Robust and Versatile Nanosystem for αvβ3-Integrin Targeting

**DOI:** 10.3389/fchem.2021.690357

**Published:** 2021-05-28

**Authors:** Angelina Sacchi, Anna Maria Gasparri, Matteo Monieri, Giulia Anderluzzi, Barbara Colombo, Alessandro Gori, Angelo Corti, Flavio Curnis

**Affiliations:** ^1^ Tumor Biology and Vascular Targeting Unit, Division of Experimental Oncology, IRCCS San Raffaele Scientific Institute, Milan, Italy; ^2^ Università Vita-Salute San Raffaele, Milan, Italy; ^3^ Istituto di Scienze e Tecnologie Chimiche, C.N.R., Milan, Italy

**Keywords:** isoAsp-Gly-Arg (isoDGR), αvβ3 integrin, TNF, gold nanoparticles, lipoamide, tumor vascular targeting, polyethylene glycol

## Abstract

Gold nanoparticles functionalized with *iso*DGR, a tripeptide motif that recognizes αvβ3 integrin overexpressed in tumor vessels, have been used as nano-vectors for the delivery of cytokines to tumors. Functionalization of nanogold with this peptide has been achieved by coating nanoparticles with a peptide-albumin conjugate consisting of heterogeneous molecules with a variable number of linkers and peptides. To reduce nanodrug heterogeneity we have designed, produced and preclinically evaluated a homogeneous and well-defined reagent for nanogold functionalization, consisting of a head-to-tail cyclized CG*iso*DGRG peptide (*iso1*) coupled *via* its thiol group to maleimide-PEG_11_-lipoamide (LPA). The resulting *iso1*-PEG_11_-LPA compound can react with nanogold *via* lipoamide to form a stable bond. *In vitro* studies have shown that *iso1*, after coupling to nanogold, maintains its capability to bind purified αvβ3 and αvβ3-expressing cells. Nanogold functionalized with this peptide can also be loaded with bioactive tumor necrosis factor-α (TNF) to form a bi-functional nanodrug that can be stored for three days at 37°C or >1 year at low temperatures with no loss αvβ3-binding properties and TNF-cytolytic activity. Nanoparticles functionalized with both *iso1* and TNF induced tumor eradication in WEHI-164 fibrosarcoma-bearing mice more efficiently than nanoparticles lacking the *iso1* targeting moiety. These results suggest that *iso1*-PEG_11_-LPA is an efficient and well-defined reagent that can be used to produce robust and more homogeneous nano-vectors for the delivery of TNF and other cytokines to αvβ3 positive cells.

## Introduction

Colloidal gold is a well-tolerated nanomaterial currently exploited for several applications in the field of nanomedicine (Cai et al., 2008; Giljohann et al., 2010). For example, gold nanoparticles have been exploited for various cancer treatment modalities, including tumor photothermal ablation therapy, radiosensitization, tumor imaging, and drug delivery (Frederix et al., 2003; Anker et al., 2008; Cai et al., 2008; Jain et al., 2008; Murphy et al., 2008; Vines et al., 2019). In particular, recent studies have shown that gold nanoparticles can be used as nano-vectors for delivering cytokines to tumors and, consequently, for enhancing their therapeutic index, such as in the case of tumor necrosis factor-α (TNF) (Paciotti et al., 2004; Farma et al., 2007; Libutti et al., 2010; Powell et al., 2010; Shenoi et al., 2013; Koonce et al., 2015; Paciotti et al., 2016). In this setting, the improved activity of cytokines is likely dependent on a “passive” targeting mechanism owing to the presence of abnormally leaky vasculature in tumors, which leads to the so-called “*enhanced permeability and retention* (EPR)” effect of nanoparticles in neoplastic tissues (Matsumura and Maeda, 1986; Chithrani et al., 2006).

We have previously shown that peptides containing *iso*Asp-Gly-Arg (*iso*DGR), a tripeptide motif that recognizes the αvβ3 integrin overexpressed in tumor vessels and on different tumor cell types (Avraamides et al., 2008; Desgrosellier and Cheresh, 2010), can be exploited as ligands for targeted delivery of various compounds to tumors, including drugs, imaging compounds and nanoparticles (Curnis et al., 2008; Curnis et al., 2013; Corti et al., 2017; Nardelli et al., 2018). In particular, we have shown that a cyclic CG*iso*DGRG peptide (called *iso1*) coupled to human serum albumin (*iso1*-HSA) can be used for the functionalization of nanogold bearing TNF or interleukin-12 (IL12), to enable “*active*” targeted delivery of nanoparticles to the tumor vasculature (Curnis et al., 2013; Gasparri et al., 2019). Studies in tumor-bearing mice have shown that extremely low doses of these nanoparticles (*iso1*-HSA/Au/TNF and *iso1*-HSA/Au/IL12) can deliver pharmacologically active doses of cytokines to murine tumors, with no evidence of toxicity, whereas nanoparticles lacking *iso*DGR were inactive, in line with the hypothesis that *iso*DGR could indeed contribute to cytokine delivery through an “*active*” targeting mechanism.

Despite the promising results obtained with *iso1*-HSA/Au/TNF and *iso1-*HSA*/*Au/IL12 as anti-cancer agents, these formulations have important drawbacks related to the use of *iso1*-HSA for nanogold functionalization with *iso*DGR. First, this peptide-protein conjugate, which consists of albumin molecules with a variable number of linkers (6-7 linkers) and peptides (4-5 isoDGR peptides) per molecule (Curnis et al., 2013), leads to the formation of heterogeneous nanoparticles. Second, the HSA used to prepare *iso1*-HSA may consist of different albumin isoforms (Kragh-Hansen et al., 2013), thereby representing an additional source of molecular heterogeneity. Third, considering that HSA is purified from human plasma donors, the isoform composition of different lots may also vary. Thus, all these issues represent important drawbacks in using *iso1*-HSA as a reagent for nanogold functionalization, since they may have an impact on product manufacturing, analysis, lot-to-lot consistency, pharmacology, and toxicology.

To overcome these problems, we tested the feasibility of an alternative strategy for nanogold functionalization with isoDGR based on the use of a homogeneous and well-defined reagent, consisting of a peptide-polyethyleneglycol-lipoamide conjugate (*iso1*-PEG_11_-LPA), instead of *iso*1-HSA. We show that gold nanoparticles functionalized with this compound maintain their αvβ3 recognition properties and are stable. Furthermore, we provide experimental evidence that these nanoparticles can be used as carriers for delivering TNF to tumors and to induce more efficient anti-tumor effects than nanoparticles lacking the isoDGR targeting moiety.

## Materials and Methods

### Reagents

Human serum albumin (HSA) (Baxter); human natural αvβ3 integrin (Immunological Science); anti-polyethyleneglycol (PEG) monoclonal antibody (mAb), clone 26A04 (Abcam); bovine serum albumin (BSA) fraction-V (Sigma); gold nanoparticles (25 nm, A_520 nm_ ∼1 unit/ml) (Aurion); MAL-dPEG_11_-lipoamide (Mal-PEG_11_-LPA) and MAL-dPEG_3_-lipoamide (Mal-PEG_3_-LPA) (Quanta Biodesign). Murine TNF was prepared as described previously (Curnis et al., 2013).

### Peptide Synthesis and Characterization

**FIGURE 1:**
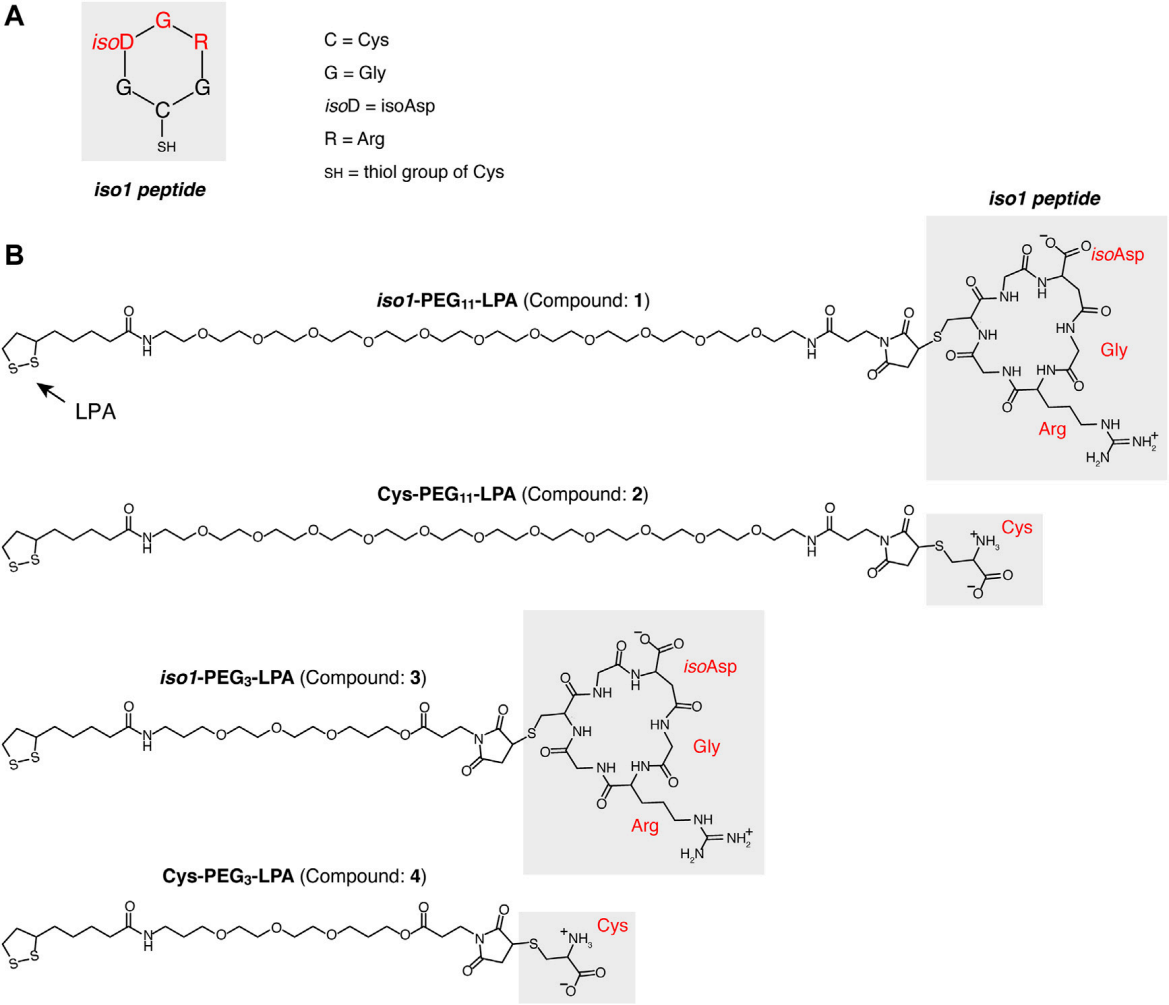
Structure of compound **1, 2, 3** and **4**. **(A)** Schematic representation of the CG*iso*DGRG head-to-tail cyclized peptide (*iso1*). **(B)** Structures of conjugates consisting of *iso1* or *Cys* coupled with MAL-PEG_11_-lipoamide or MAL-PEG_3_-lipoamide to form *iso1*-PEG_11_-LPA (compound **1**), *Cys*-PEG_11_-LPA, (compound **2**), *iso1*-PEG_3_-LPA (compound **3**), *Cys*-PEG_3_-LPA (compound **4**), respectively.

Linear peptide CG*iso*DGRG was assembled using the Fmoc-based solid-phase method (Fields and Noble, 1990) on a 2-CTC resin. Upon synthesis, the peptide was cleaved from the resin, using 1% trifluoroacetic acid (TFA) in dichloromethane, and dried. The resulting material was dissolved in *N,N*′-dimethylformamide (DMF, 50 mM) and treated with HBTU/DIEA (1 eq.) to obtain the fully protected cyclo-CGisoDGRG peptide. DMF was removed under vacuum and the resulting crude material was directly treated with TFA-based cleavage mixture (TFA 95%, TIS 2.5%, Thianisole 2.5%) to obtain the unprotected peptide, which was recovered by precipitation in cold diethyl ether. The peptide was purified by reverse-phase (RP)-HPLC, using a Shimpack GWS C18 column (10 µm, 21.2 mm x 250 mm, Shimadzu), lyophilized, and stored at −80°C. Aliquots of the product were dissolved in water and stored at −80°C until use. The identity and purity of the product, called *iso1* (*see* Figure 1), were confirmed by electrospray ionization mass spectrometry [expected monoisotopic mass (MH^+^): 546.22 Da; found: 546.27 Da] and RP-HPLC analysis (purity > 95%).

### Preparation of *iso1*-PEG_11_-LPA, *Cys*-PEG_11_-LPA, *iso1*-PEG_3_-LPA, and *Cys*-PEG_3_LPA Conjugates

*Iso1*-PEG_11_-LPA was prepared by coupling *iso1* to Mal-PEG_11_-LPA using a 1:1.2 molar ratio, as follows: 4 mg of *iso1* in 100 µl of 0.4 M sodium phosphate buffer, pH 7.8, were mixed with 50 µl of acetonitrile (final pH: ∼7). The resulting solution was chilled on ice and mixed with 7.7 mg of MAL-PEG_11_-LPA in 100 µl of 50% acetonitrile and left to react under gentle shaking at room temperature. The coupling reaction was monitored by RP-HPLC and stopped when complete reagent conversion occurred. In parallel, a control conjugate was prepared using cysteine in place of *iso1*. Coupling reactions were monitored by RP-HPLC using a Shimpack GWS C18 column (5 µm, 4.6 mm x 150 mm, Shimadzu) connected to Shimadzu Prominence HPLC (mobile *phase A*, 0.1% trifluoroacetic acid in water; mobile *phase B*, 70% acetonitrile, 0.1% trifluoroacetic; linear gradient 10–100% B; in 20 min; flow rate, 1 ml/min). The final products were purified by RP-HPLC using a Jupiter C18 column (10 µm, 21.2 mm x 250 mm, Phenomenex), and the same mobile phases indicated above (liner gradient, 0–90% B, 45 min; flow rate, 14 ml/min). The products were lyophilized, resuspended in water, and stored at −20°C. Furthermore, *iso1* and Cys were also coupled to MAL-dPEG_3_-LPA, i.e., a compound with a shorter PEG chain, using the same procedures. The identity of each conjugate [*iso1*-PEG_11_-LPA (compound **1**), Cys-PEG_11_-LPA (compound **2**), *iso1*-PEG_3_-LPA (compound **3**), and Cys-PEG_3_LPA (compound **4**)] was checked by mass spectrometry analysis using an LTQ-XL Orbitrap mass spectrometer (Thermo Fischer) (Supplementary Table S1 and Supplementary Figure S1).

### Nanogold Functionalization With Compound 1

To functionalize gold nanoparticles with *iso1* we mixed aliquots of colloidal gold (1 ml, adjusted to pH ∼7.5 with sodium hydroxide) with 4–32 µg of compound **1** in 5 mM sodium phosphate buffer, pH 7.4 (100 µl). The mixture (final pH ∼7.5) was incubated at room temperature for 2 h. To saturate gold nanoparticles, we then added 0.5% HSA (in 25 µl aliquots every 2 min, for four times) and left to incubate for 10 min at room temperature. The product was then centrifuged at 13,000x g for 15 min. The pellets were resuspended in 5 mM sodium phosphate buffer, pH 7.33, containing 0.05% HSA (*buffer A*). The centrifugation/washing steps were repeated twice. The final product (called **1**-Au) was resuspended with 1 ml of buffer A and stored at 4°C.

### Nanogold Functionalization With Compound 1 and TNF

Bifunctional gold NPs bearing compound **1** and TNF (called **1**-Au/TNF) were prepared as follows: a solution containing 0.540 mg of compound **1** and 2.88 mg of TNF in 18 ml of 5 mM sodium phosphate, pH 7.4, was slowly added (1 ml/min) to 180 ml of 25 nm-nanogold, with pH adjusted to ∼7.5 with sodium hydroxide, and left to incubate for 40 min at room temperature under shaking. The product was mixed with 18 ml of 0.5% HSA in water (slowly added) and left to incubate for an additional 15 min, to saturate gold NPs. The mixture was centrifuged (13,000x g for 30 min) and resuspended in buffer A (three times). The pellet was then resuspended with 1.8 ml of buffer A, filtered (0.22 µm pore size, Millex-GV Filter), and stored at −80°C. Control nanoparticles bearing compound **2** and TNF (called **2**-Au/TNF) were prepared following the above procedure, except that in this case, 0.360 mg of compound **2** was used.

### Physicochemical Characterization of Nanoparticles

Absorption spectra of coated- or uncoated-NPs were recorded using an UltroSpec 2100 spectrophotometer (Amersham Biosciences), a 1 cm path-length quartz cuvette, and *buffer A* or 5 mM sodium citrate buffer, pH 6.0, respectively, as blanks. The concentration of coated-NPs was calculated by interpolating the absorbance values at 530 nm on a calibration curve obtained using uncoated nanogold (stock solution: 3.3 × 10^11^ NPs/ml, A_530_ nm: 0.96 U/ml).

Transmission electron microscopy (TEM) analysis was performed using a TALOS L120C microscope (ThermoScientific) as described previously (Gasparri et al., 2019). Morphometric analysis of nanoparticle’s shape and diameter was performed using the ImageJ software, essentially as previously reported (Rice et al., 2013).

### Binding of Nanogold Functionalized With Compound 1 or 2 to Melanoma Cells

Human MSR3 melanoma cells were grown in Iscove’s modified Dulbecco’s medium (IMDM) (Lonza) supplemented with heat-inactivated 10% fetal bovine serum (FBS), 2 mM L-glutamine, and 1% penicillin/streptomycin. Cells were seeded into a 96-well plate (20,000 cells/well) and incubated for 24 h in complete cell culture medium. After medium removal, the cells were incubated with nanogold functionalized with compound **1** or **2** in cell culture medium (1.7 × 10^11^ NPs/ml, 100 µl/well, 1.5 h at 37°C, 5% CO_2_). After washing, the cells were further incubated for 4 h at 37°C, 5% CO_2_, and then photographed using a bright field microscopy.

### αvβ3 Integrin and Anti-PEG Antibody Binding Assays

The bifunctional properties of **1**-Au/TNF were checked using two sandwich assays based on αvβ3-and anti-PEG antibody-coated plates followed by an anti-TNF polyclonal antibody. The αvβ3/anti-TNF polyclonal antibody sandwich assay was performed essentially as described (Gasparri et al., 2019). Briefly, various amounts of nanoparticles in 25 mm Tris-HCl, pH 7.4, containing 150 mM sodium chloride, 1 mM magnesium chloride, 1 mM manganese chloride, 1% w/v BSA (*binding buffer*), were added to microtiter plates coated with or without αvβ3 (0.5–1 µg/ml). The binding of nanoparticles was then detected using a rabbit anti-TNF polyclonal antiserum (1:1,000), followed by a polyclonal goat anti-rabbit HRP-conjugate. Bound peroxidase was detected by adding the *o*-phenylenediamine chromogenic substrate. The anti-PEG antibody/anti-TNF polyclonal antibody sandwich assay was carried out essentially as described above using a microtiter plate coated without or with an anti-PEG mAb (5 µg/ml) in the capture step, and the anti-TNF polyclonal antibody in the detection step.

### *In Vitro* TNF Bioassay

The amount of bioactive TNF bound to gold NPs was determined using an *in vitro* bioassay based on TNF-induced cytolysis of murine L-M fibroblasts, as described (Curnis et al., 2013), except that cell viability was quantified using the PrestoBlue Cell Viability (Invitrogen). International murine TNF reference standard (NIBSC, ID: 88/532) was used to calibrate the assay.

### *In Vivo* Studies

All procedures on mice were approved by the San Raffaele Institutional Animal Care and Use Committee, according to institutional guidelines, and in compliance with national (D.L. N.26, 04/03/2014) and international law and policies (new directive 2010/63/EU). Murine WEHI-164 fibrosarcoma cells (ATCC, cat. CRL-1751) were cultured in DMEM supplemented with 10% fetal bovine serum, 2 mM glutamine, 50 μg/ml streptomycin, 100 U/ml penicillin, and 0.25 μg/ml amphotericin-B. WEHI-164 cells were tested for *mycoplasma* contamination using the *Mycoplasmacheck* testing service provided by Eurofins Genomics before their use *in vivo.* BALB/c mice (6–8 weeks old, Charles River Laboratories), weighing 18–20 g, were challenged with subcutaneous injection in the left flank with 1.5 × 10^6^ WEHI-164 cells. Six days later, mice were injected (i.v.) with nanodrugs in 0.9% sodium chloride. Tumor growth was monitored by measuring the tumor size with a caliper. Tumor volumes were estimated by calculating r1 × r2 × r3 × 4/3π, where r1 and r2 are the longitudinal and lateral radii, and r3 is the thickness of the tumor protruding from the surface of normal skin. Animals were sacrificed before tumors reached a volume of 1 cm^3^. Tumor sizes are shown as mean ± SE.

## Results

### Preparation of *iso1*-PEG-LPA Conjugates

To have at hand an *iso*DGR peptide that can be directly attached to gold nanoparticles, we have coupled cyclo-CG*iso*DGRG (*iso1*) to MAL-PEG_11_-lipoamide and MAL-PEG_3_-lipoamide, two cross-linking reagents with different polyethylene glycol chains (Figure 1). Both reagents contain a maleimide group (MAL), which can form a thioether bond with the sulfhydryl group of *iso1*, and a lipoamide group that can react with nanogold to form dative bonds. In parallel, we prepared control ligands consisting of cysteine in place of *iso1*. These conjugates, called *iso1*-PEG_11_-LAP (*compound* **1**), Cys-PEG_11_-LAP (*compound* **2**), *iso1*-PEG_3_-LAP (*compound* **3**), and Cys-PEG_3_-LAP (*compound* **4**), were purified by RP-HPLC. The identity of each product was confirmed by mass spectrometry analysis (Supplementary Table S1 and Supplementary Figure S1).

### Identification of the Optimal *iso1*-PEG-LPA Compound for Nanogold Functionalization

**FIGURE 2:**
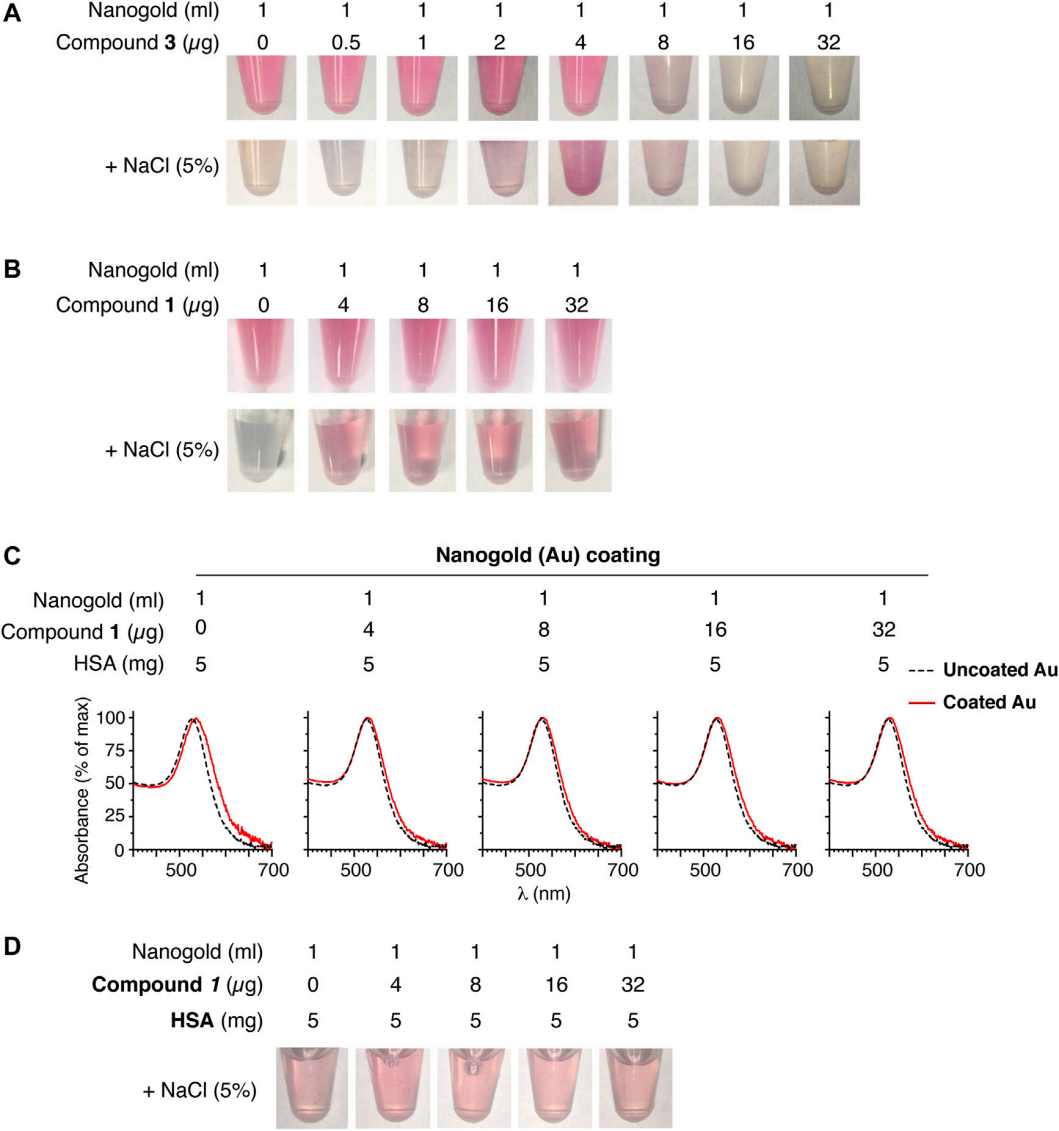
Compound **1** protects nanogold from aggregation better than compound **3**. **(A, B)** Representative photographs of nanogold (25-nm, ∼1 U/ml at A_520_ nm) mixed with the indicated amount of compound **3** or **1** before and after the addition of sodium chloride (5% final). Marked color change from red to gray is an indication of nanoparticle aggregation. **(C)** UV-Vis spectra of gold nanoparticles uncoated (*dashed line*) or coated (*solid red line)* with the indicated amounts of compound **1** and human serum albumin (HSA, added as a second stabilizer). **(D)** Effect of sodium chloride (5% final concentration) on aliquots of the products shown in **(C)**. Note that all products display similar colors, suggesting good nanoparticle stability.

To functionalize nanogold with isoDGR, we then mixed 25 nm-gold nanoparticles with various concentrations of compound **1** or **3** and left them to incubate for 2 h. We observed nanogold aggregation with compound **3** at concentrations > 4 µg/ml, as indicated by a marked change of nanogold color, but not with compound **1** (Figures 2A,B, upper panels). Although compound **3** did not cause nanogold aggregation at concentrations < 4 µg/ml, this compound was unable to inhibit the aggregation induced by the addition of 5% (w/v) NaCl (Figure 2A, lower panels). This indicates that compound **3** loaded on gold nanoparticles was not sufficient to inhibit salt-induced aggregation. In contrast, all the tested doses of compound **1** (4–32 µg/ml) did not cause nanogold aggregation by itself and could efficiently protect nanoparticles from salt-induced aggregation (Figure 2B, lower panels). Based on this finding, nanogold functionalized with compound **1** and its relevant control (compound **2,** lacking the *iso1*) were selected for further studies, whereas nanogold functionalized with compounds **3** and **4** were not further investigated. Nanoparticles prepared with compound **1** were further stabilized with human serum albumin and characterized by UV-Vis spectrophotometric analysis. The results showed that these nanoparticles were homogeneous, not aggregated, and resistant to salt-induced aggregation (Figures 2C,D).

### Binding of Gold Nanoparticles Functionalized With Compound 1 (1-Au) or 2 (2-Au) to MSR3 Cells

**FIGURE 3:**
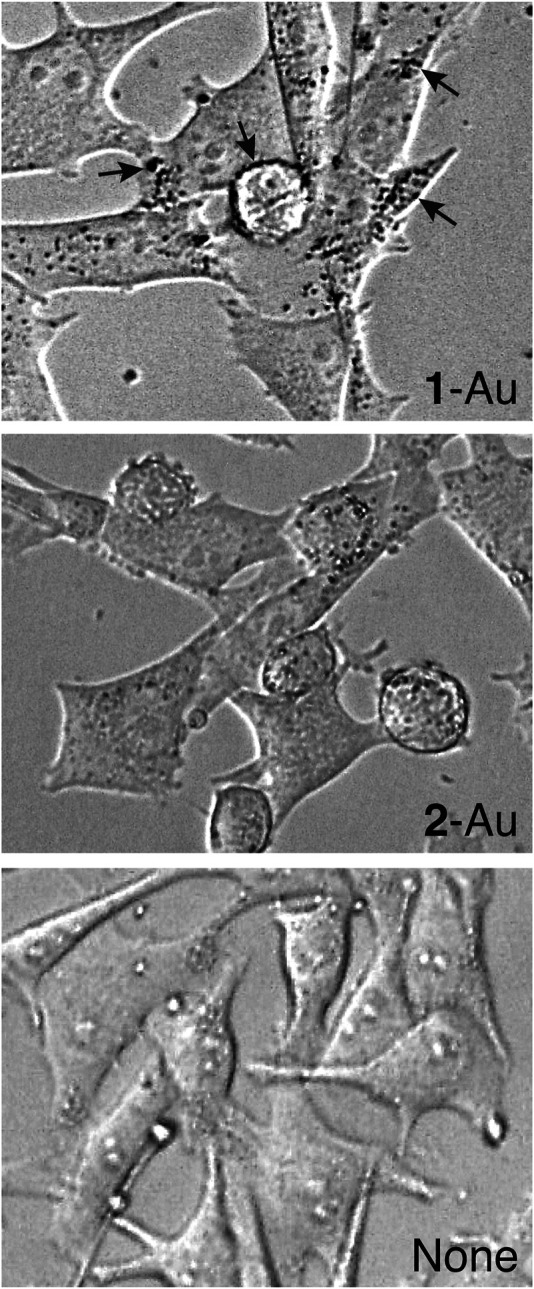
Binding of gold nanoparticles functionalized with compound **1** (**1**-Au) or compound **2** (**2**-Au) to MSR3 cells. Phase-contrast microscopic images of MSR3 cells after incubation with or without **1**-Au or **2**-Au. **1**-Au and **2**-Au were prepared using 4 µg/ml of each compound per ml of gold sol. *Arrows* indicate black dots corresponding to nanoparticle agglomerates bound to cells. Magnification, 40X.

To assess whether nanogold functionalized with compound **1** (called **1**-Au) could bind membrane-associated αvβ3, we tested the binding of this product to MSR3 cells, a human melanoma cell line that expresses high levels of αvβ3 and previously used for the characterization of *iso1* (Nardelli et al., 2018). In parallel, control nanogold functionalized with compound **2** (**2**-Au) was also tested. Phase-contrast microscopy experiments showed granular dot-like staining in MRS cells incubated with **1**-Au, but little, or not at all, with **2**-Au (Figure 3), suggesting that the binding of **1**-Au was mediated by *iso1*. These data are in line with the hypothesis that **1**-Au can recognize the αvβ3 integrin expressed on the cell surface.

### Preparation and Characterization of 1-Au/TNF

To demonstrate that compound **1** can be exploited for delivering nanogold to αvβ3-positive tumors, we coated gold NPs with compound **1** and murine TNF, a cytokine endowed with potent anti-tumor activity. Optimal nanogold loading with cytokine and ligand was achieved with 16 µg/ml of TNF and 3–4 µg/ml of compound **1** or 2–4 µg/ml of compound **2** (Supplementary Figures S2–S4 and see also Supplementary Material). Based on these results, we prepared a larger batch of bifunctional nanoparticles functionalized with isoDGR and TNF using 16 µg/ml of TNF and 3 µg/ml of compound **1** (**1**-Au/TNF), a larger batch of control nanoparticles lacking isoDGR using 16 µg/ml of TNF and 2 µg/ml of compound **2** (**2**-Au/TNF).

**FIGURE 4:**
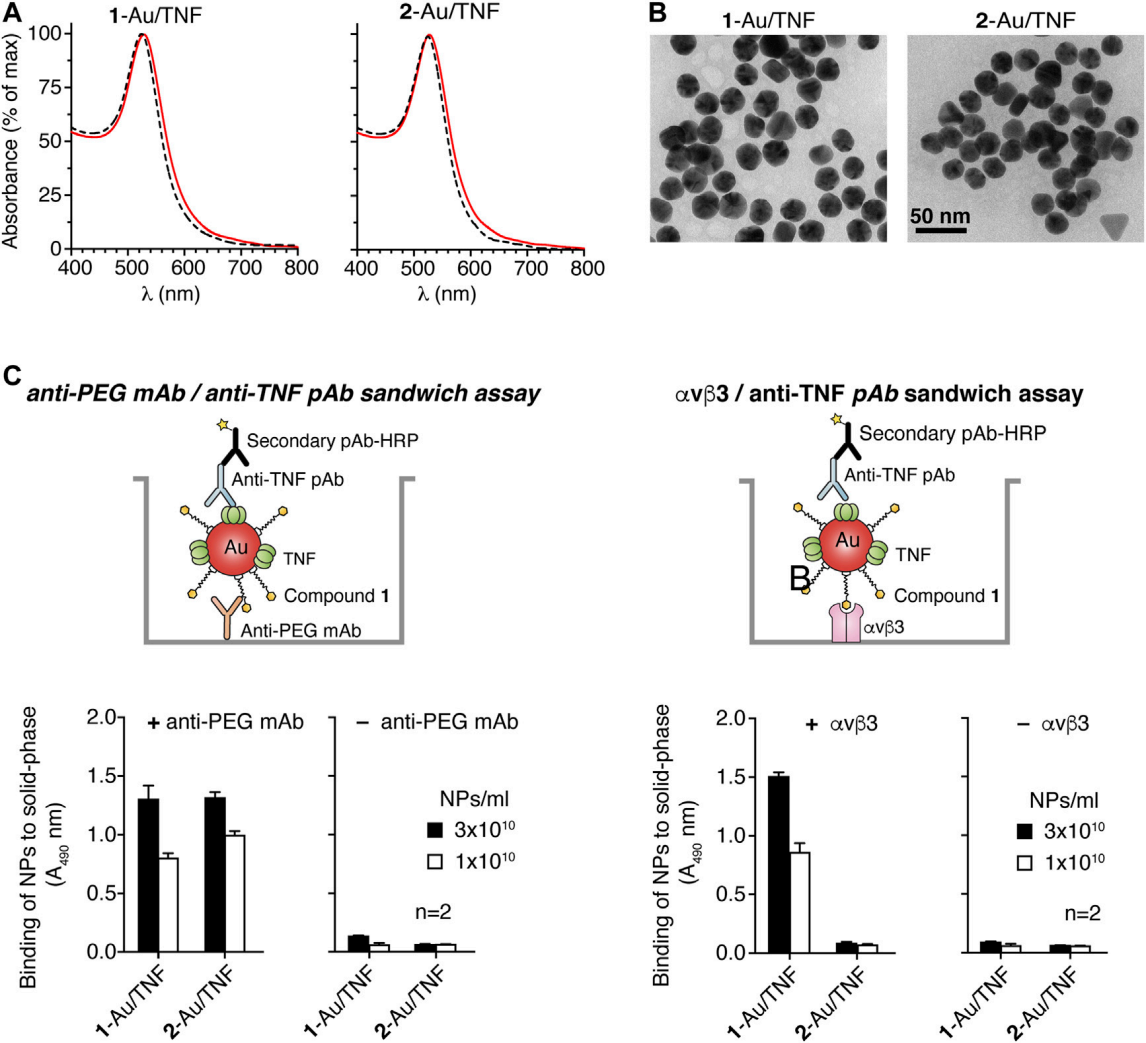
Characterization of **1**-Au/TNF and **2**-Au/TNF nanoparticles. **(A)** UV-Vis absorption spectra of nanogolds. *The dotted line* corresponds to uncoated 25 nm gold nanoparticles. **(B)** Transmission electron microscopy (TEM) of **1**-Au/TNF and **2**-Au/TNF. Representative microphotographs of **1**-Au/TNF and **2**-Au/TNF are shown. Morphometric analysis of nanoparticles shows that **1**-Au/TNF and **2**-Au/TNF consist of gold nanospheres with maximal diameters of 25.6 ± 2.3 nm and 26.7 ± 2.2 nm (mean ± SD, *n* = 100 NPs), respectively, and with a roundness value of 0.87 ± 0.07 and 0.090 ± 0.05, respectively (a roundness value of 1 corresponds to a perfect circle). **(C)** Binding of nanoparticles to microtiter plates coated with (+) or without (–) an anti-PEG mAb or αvβ3 integrin, as detected with an anti-TNF polyclonal antibody and HRP-labeled goat anti-rabbit antiserum. The results of a representative experiment are shown. *Bars*, mean ± SE of technical duplicate.

UV-Vis spectrophotometric analysis of both products showed single absorption peaks at 530 nm with a similar width, indicating that both products contained low (undetectable) amounts of aggregates (Figure 4A and Table 1). Transmission electron microscopy showed that both nanodrugs had a maximal diameter of about 28 nm and were composed by spherical nanoparticles (Figure 4B).

**TABLE 1:** Characterization of 1-Au/TNF and 2-Au/TNF by UV-visible spectroscopy (UV-Vis) and cytotoxicity assays.

Nanodrug	UV-vis^a^				TNF-bioassay^c^	
	*λ*_max_ (nm)	*PW 75%*	A_650_/A_530_	NPs/ml	µg/ml	Molecules/NP
**1**-Au/TNF	528 ± 0.25 (*n = 2*)^b^	55.3 ± 2.0 (*n = 2*)	0.096 ± 0.021 (*n = 2*)	2.21 (±0.095) × 10^13^ (*n = 3*)	20.4 ± 4.9 (*n = 4*)	10.7 ± 2.5 (*n = 4*)
**2**-Au/TNF	527	54	0.074	3.42 × 10^13^	41.1 ± 9.4 (*n = 4*)	13.7 ± 3.1 (*n = 4*)
Uncoated Au	524 ± 1.0 (*n = 2*)	49.7 ± 0.6. (*n = 2*)	0.052 ± 0.025 (*n = 2*)	3.30 × 10^11^	-	-

a *Λmax*: wavelength of peak absorbance; *PW 75%:* peak-width at 75% of height; A_650_ nm/A_530_ nm: absorbance ratio. Mean ± SD.

b *n*, number of independent experiments.

c As determined by L-M cell cytotoxic assay using the recombinant TNF international standard (mean ± SE).

To assess the presence of compound **1** or **2** and TNF on the two nanodrugs, we measured the capability of each product to form molecular sandwiches with an anti-PEG monoclonal antibody (mAb) and an anti-TNF polyclonal antibody (pAb), or with αvβ3 and anti-TNF pAb. To this aim, we performed assays based on the use of microtiter plates coated with anti-PEG mAb or αvβ3 in the capture step and anti-TNF pAb in the detection step (*see* Figure 4C *for a schematic representation of the assays*). **1**-Au/TNF and **2**-Au/TNF showed comparable binding properties when anti-PEG mAb-coated plates were used (Figure 4C, *left panel*), suggesting that these nanodrugs were loaded with similar amounts of PEG-containing ligands and TNF. In contrast, only **1**-Au/TNF was detected when αvβ3 was used in place of anti-PEG mAb in the capture step, as expected (Figure 4C, *right panel*). Of note NPs functionalized with compound **3** (a ligand characterized by a shorter PEG length) and stabilized with TNF to prevent aggregation (**3**-Au/TNF) bound αvβ3 less efficiently than **1**-Au/TNF (Supplementary Figure S5), suggesting that the PEG_11_ chain of **1**-Au/TNF is important for the nanodrug/αvβ3 interaction.

To quantify the amount of bioactive TNF loaded onto both nanodrugs, we then tested their cytotoxic effects against murine L-M fibroblasts, using TNF as a reference standard. Based on the biological effects observed, we estimate that the potency of each nanoparticle of **1** *-*Au/TNF and **2** *-*Au/TNF was equivalent to ∼11 and ∼14 TNF molecules, respectively (Table 1), suggesting that both conjugates were loaded with similar amounts of bioactive TNF. Overall, these and the above results suggest that the functionalization of nanogold with lipoamide-*iso*DGR peptide and TNF is feasible.

#### Stability of **1**-Au/TNF

**FIGURE 5:**
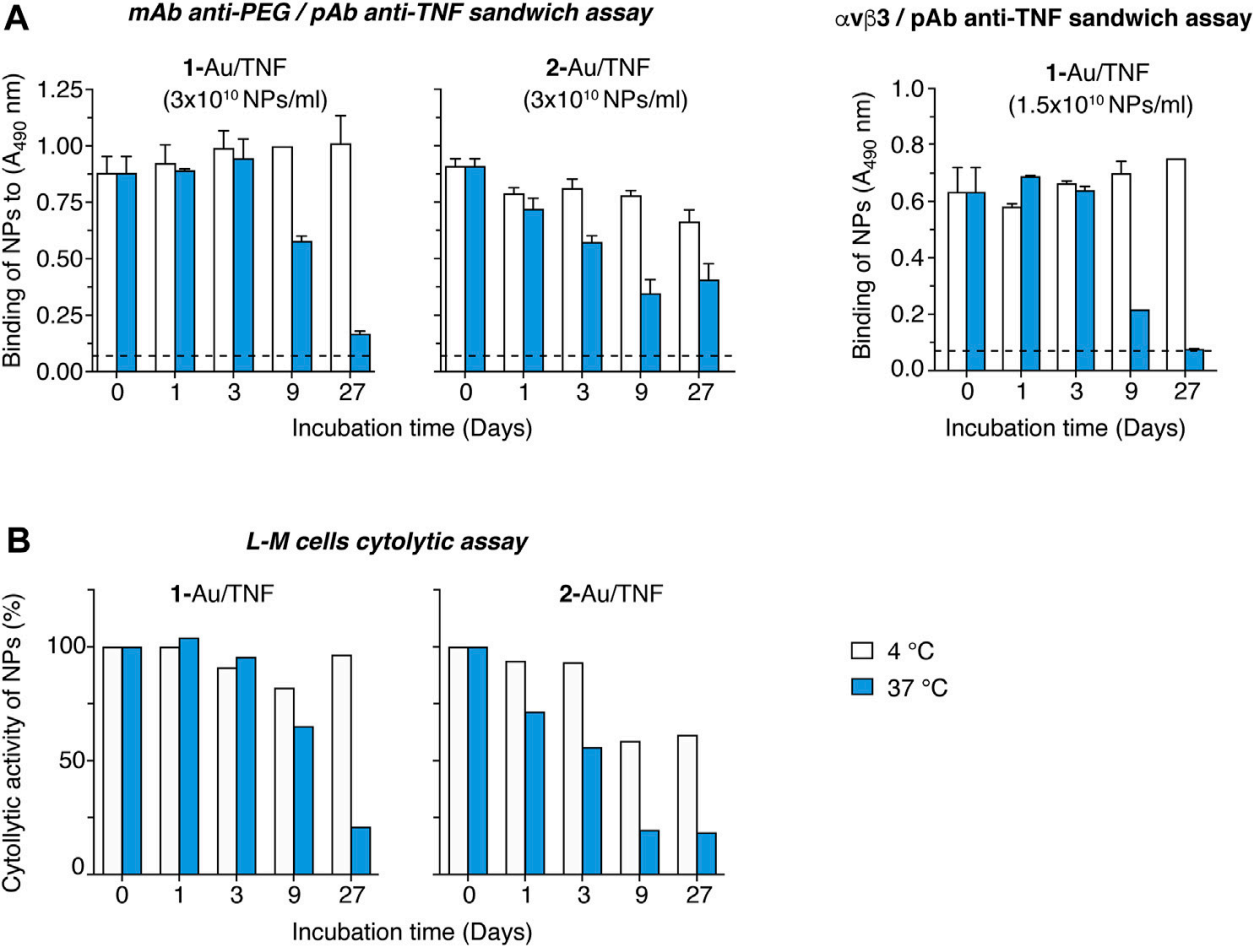
Stability studies of **1**-Au/TNF and **2**-Au/TNF. **1**-Au/TNF and **2**-Au/TNF, in storage buffer (5 mM phosphate buffer pH 7.33, 0.05% HSA), were left to incubate at 4°C or 37°C for the indicated time and tested using the assays indicated on each panel. **(A)** Binding of nanodrugs to microtiter plates coated with an anti-PEG mAb (*left panels*) or αvβ3 (*right panel*), as detected with a polyclonal antibody anti-TNF followed by HRP-labeled goat anti-rabbit antiserum (*see* Figure 4, for a schematic representation of the sandwich assays). *Bars*, mean ± SE of technical duplicate. **(B)** Cytotoxic effects of nanodrugs on L-M cells. L-M cells (30,000 cells/well) were cultured in complete medium supplemented with 2 µg/ml actinomycin D and in the presence of various amounts of nanodrugs, for 20 h at 37°C, 5% CO_2_. Cell viability was quantified using the PrestoBlue cell viability reagent.

The stability of **1**-Au/TNF in the storage buffer (5 mM phosphate buffer containing 0.05% HSA) was then investigated. To this aim, **1**-Au/TNF and **2**-Au/TNF were left to incubate at 4°C or 37°C for various times (from 1 to 27 days) and then analyzed using the anti-PEG mAb/anti-TNF pAb and αvβ3/anti-TNF pAb sandwich assays. No significant changes occurred upon nanodrug storage for 9 days at 4°C or 3 days at 37°C (Figure 5A). In contrast, both products showed a progressive decline in their binding properties upon storage at 37°C for longer times (Figure 5A). Furthermore, no change in the biological activity of both nanodrugs was observed upon storage up to 3 days at 4°C (Figure 5B). These results suggest that nanogold functionalized with TNF and *iso1* (the latter *via* lipoamide) can be stored at 4°C for at least 27 days without loss of function. Furthermore, no significant changes occurred upon nanodrug storage for > 1 year at –80°C (Supplementary Figure S6).

#### Release of TNF From 1-Au/TNF in Cell Culture Medium

**FIGURE 6:**
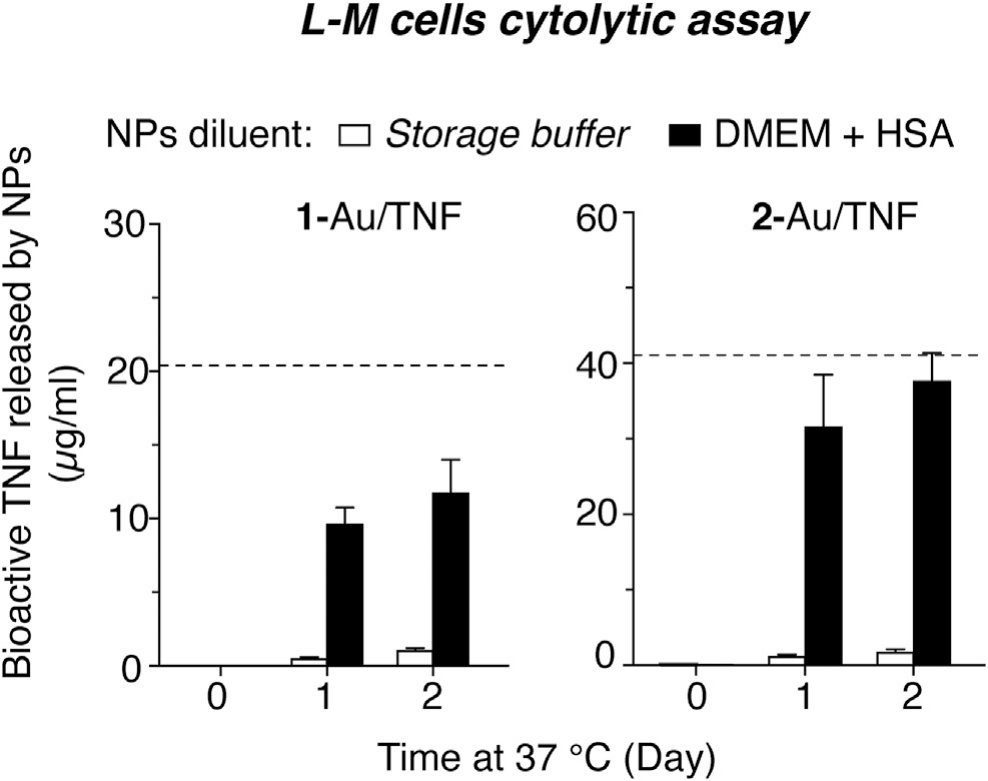
Release of biologically active TNF from **1**-Au/TNF and **2**-Au/TNF upon incubation in cell culture medium. Aliquots of **1**-Au/TNF and **2**-Au/TNF (1-3x10^11^ NPs/ml, 100 µl) were centrifuged, resuspended in 5 mM phosphate buffer, pH 7.33, containing 0.5 mg/ml HSA (*storage buffer*) or in DMEM containing 0.5 mg/ml HSA and left to incubate at 37°C for the indicated time. After centrifugation, the supernatants were collected. The amount of bioactive TNF released in the supernatant at various time points was then measured using a cytotoxicity assay based on L-M cells. *Bars* represent the concentration of bioactive TNF found in the supernatant (mean ± SEM, of 4 technical replicates); the *dashed lines* represent the concentration of total TNF (bound and released).

The stability of nanodrugs in DMEM cell culture medium, i.e., in conditions used in *in vitro* cytotoxicity assays, was also investigated. To this aim, **1**-Au/TNF and **2**-Au/TNF were centrifuged, resuspended in DMEM containing 0.5 mg/ml HSA, and left to incubate at 37°C for 1 and 2 days. The amount of bioactive TNF released in the supernatant was measured, after further NPs centrifugation, by cytotoxicity assays. The results show that about 50 and 80% of the bioactive TNF bound to NPs was released from **1**-Au/TNF and **2**-Au/TNF, respectively, after 1 day of incubation (Figure 6), suggesting that DMEM promoted the release of biologically active TNF. Considering that **2**-Au/TNF was loaded with 2-fold more TNF compared to **1**-Au/TNF (see Table 1), one possible explanation for the higher release of TNF by **2**-Au/TNF is that TNF subunits can bind to NPs in different manners depending on the molecular density on the nanogold surface. However, when the same experiment was performed with NPs in the storage buffer instead of DMEM, we observed that only a very small fraction of TNF was released from NPs in both cases (Figure 6), suggesting that in these conditions most TNF molecules remain firmly bound.

The higher release of TNF observed in DMEM suggests that part of TNF was likely released also during the cytotoxicity assays of nanodrugs, thereby contributing to their cytotoxic activity reported in Table 1.

#### Anti-Tumor Activity of 1- and 2-Au/TNF

**FIGURE 7:**
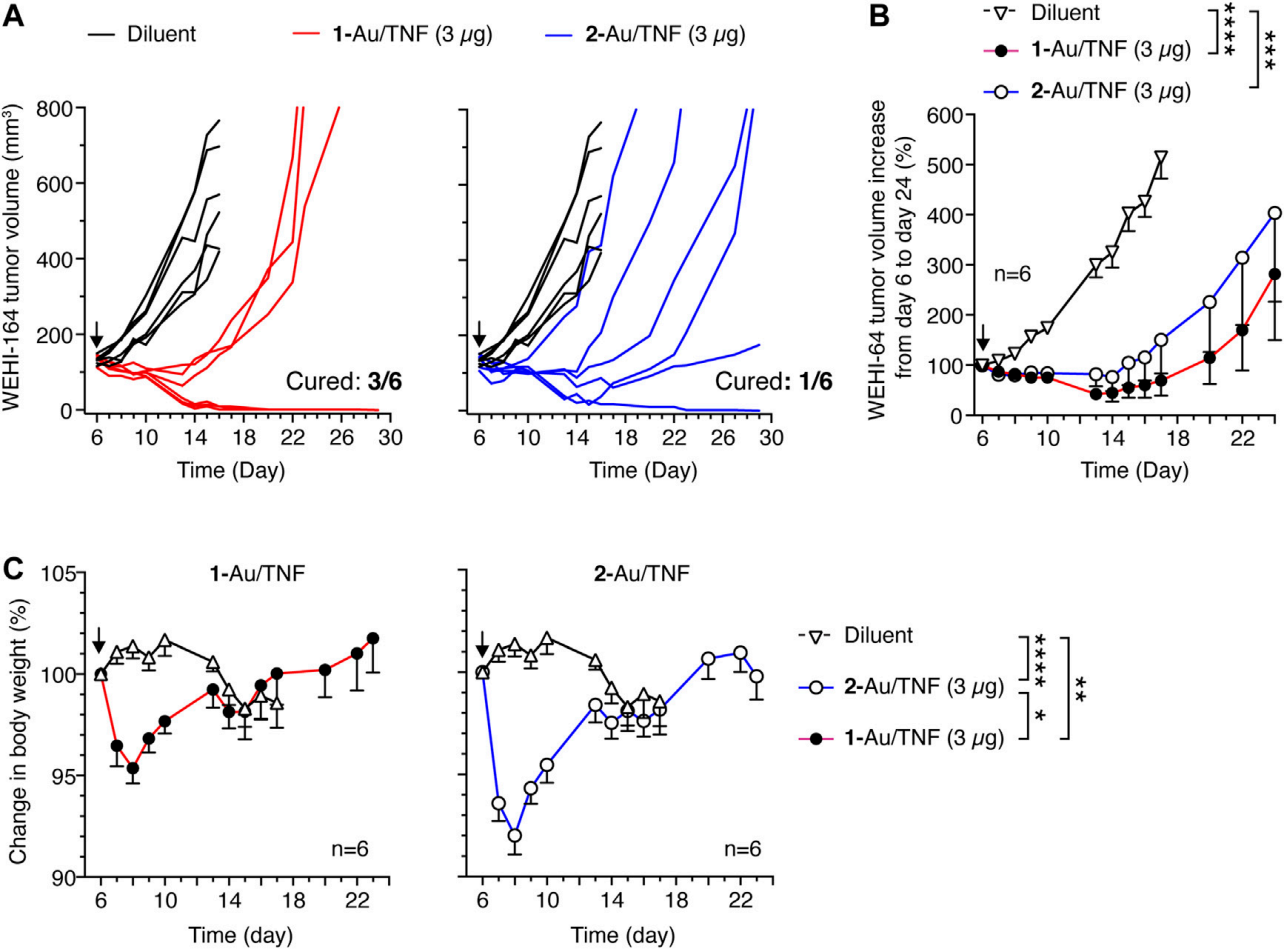
Anti-tumor effects of **1**-Au/TNF and **2**-Au/TNF in the WEHI-164 fibrosarcoma model. Tumor-bearing mice were treated at day 6 after tumor implantation with **1**-Au/TNF or **2**-Au/TNF doses equivalent to 3 µg of bioactive TNF (by L-M cytolytic assay). Nanodrugs were administered i.v. in 0.9% sodium chloride. Control mice were treated i.v. with 0.9% sodium chloride (*Diluent*). Tumor growth in each mouse **(A)** and cumulative data **(B)** are shown (mean ± SE, six mice per group). ***, *p* < 0.001; ****p < 0.0001 unpaired two-tail *t* test at day 17. **(C)** Animal body weight change after treatment (mean ± SE, six mice per group). *, *p* < 0.05; **, *p* < 0.01; ****p < 0.0001 ordinary one–way ANOVA of the area under the curve for each mouse weight (*see* Supplementary Figure S7) calculated from day 6 to day 13 with the GraphPad Prism software.

Finally, we analyzed the anti-tumor activity and toxicity of **1**-Au/TNF and **2**-Au/TNF using nanodrug doses equivalent to 3 µg of bioactive TNF, administered intravenously to immunocompetent mice bearing subcutaneous WEHI-164 fibrosarcomas. Three out of six mice (50%) were cured with **1**-Au/TNF, whereas only one out of six mice (17%) was cured with **2**-Au/TNF (Figures 7A,B), suggesting that *iso1* increased the antitumor activity to TNF-bearing nanogold. To assess the toxicity of **1**-Au/TNF and **2**-Au/TNF, we measured the loss of body weight after treatment. Notably, **1**-Au/TNF induced a lower loss of weight than **2**-Au/TNF (Figure 7C, Supplementary Figure S7), suggesting that *iso1* increased the therapeutic index (i.e., the ratio between efficacy and toxicity) of TNF-bearing nanogold, at least as judged from the loss of animal body weight.

## Discussion

In this study we have designed, produced and preclinically evaluated a new reagent for nanogold functionalization with the isoDGR motif, called *iso1*-PEG_11_-LPA. This reagent consists of head-to-tail cyclized CG*iso*DGRG peptide (*iso1*) chemically coupled *via* its thiol group to maleimide-PEG_11_-LPA cross-linking reagent. Remarkably, the coupling reaction leads to the formation of a succinimide ring between peptide and PEG_11_-LPA (*see* Figure 1). This succinimide linker is important for the functional properties of *iso1*, as in previous studies we have shown that *iso1*-HSA, a peptide-protein conjugate previously used for the functionalization of nanogold with *iso1*, contains a succinimide ring that is crucial for αvβ3 recognition (Nardelli et al., 2018). In particular, we have shown that this conjugate, produced by coupling *iso1* to human serum albumin using sulfosuccinimidyl 4-(N-maleimidomethyl)cyclohexane-1-carboxylate (sulfo-SMCC) as cross-linking reagent, has a higher affinity and selectivity for αvβ3 than *iso1* alone, thanks to the presence of the succinimide ring (Nardelli et al., 2018). NMR, computational, and biochemical studies have shown that the succinimide ring of the linker participates in αvβ3 binding through the formation of a hydrogen bond with the Tyr122β3 side chain of αvβ3 (Nardelli et al., 2018). Although the succinimide ring of the linker in *iso1*-HSA contributes to stabilizing the interactions of *iso1* with αvβ3, we have to consider that the sulfo-SMCC cross-linking reagent used for coupling *iso1* to HSA can react with different amino-groups of albumin, thereby leading to conjugate molecules bearing a variable number of linkers and peptides in different positions. Consequently, the use *iso1*-HSA for nanogold functionalization leads to heterogeneous nanoparticles. In contrast, *iso1*-PEG_11_-LPA is a homogeneous reagent (expected mass:1428.61 Da; found mass 1428.62 Da) that can react *via* lipoamide with nanogold to form a stable gold-thiol bond (Katz and Willner, 2004) and maintains the succinimide ring as a linker between *iso1* and PEG_11_-LPA. We think, therefore, that *iso1*-PEG_11_-LPA is a good candidate for replacing *iso1*-HSA for nanogold functionalization with isoDGR and for the preparation of more homogeneous nanodrugs.

This view is supported by the results of *in vitro* binding experiments showing that *iso1*, after coupling to nanogold *via iso1*-PEG_11_-LPA (herein called **1**-Au), maintained its capability to bind purified αvβ3 and to recognize MRS melanoma cells, an αvβ3-expressing cell line previously used for the characterization of isoDGR-containing compounds (Nardelli et al., 2018; Paladino et al., 2019). Gold nanoparticles functionalized with *iso1*-PEG_11_-LPA can also be loaded with ∼11 molecules/NP of biologically active TNF, demonstrating that this strategy is also suitable for preparing bi-functional nanodrugs (called **1**-Au/TNF) bearing both targeting and effector moieties.

Drug stability studies have shown that these bi-functional nanodrugs can be stored up to three days at 37°C, or >1 year at 80°C, in 5 mM sodium phosphate buffer, pH 7.33, containing 0.05% HSA, with no loss of activity in terms of αvβ3-binding and TNF-cytolytic activity. This result indicates that the new strategy used for nanogold functionalization herein described allows the production of stable nanodrugs.

The results of *in vivo* studies, performed using WEHI-164 fibrosarcoma-bearing mice, show that nanoparticles functionalized with *iso1* and TNF according to this strategy (**1**-Au/TNF) could induce tumor eradication in WEHI-164 fibrosarcoma-bearing mice more efficiently than nanoparticles lacking the *iso1* targeting moiety (**2**-Au/TNF). This result supports the hypothesis of an *active* targeting mechanism mediated by *iso1* in the case of **1**-Au/TNF. Given that WEHI-164 cells do not express αvβ3 receptors, these results also suggest that the improved anti-tumor activity of **1**-Au/TNF was mediated by targeted delivery of TNF to a tumor stroma component, likely to αvβ3-positive endothelial cells. Notably, the *iso1*-mediated targeting mechanism increased the anti-tumor activity of TNF without increasing its toxicity, as judged from the even lower loss of body weight caused by **1**-Au/TNF compared to **2**-Au/TNF. This suggests that the targeted delivery of TNF-nanogold to tumors with *iso1* can increase its therapeutic index.

In conclusion, *iso1*-PEG_11_-LPA is an efficient, well-defined, and robust reagent that can be used for nanogold functionalization with isoDGR and for enabling αvβ3 recognition by cytokine-bearing gold nanoparticles. This novel peptide-linker conjugate can overcome the need to use heterogeneous peptide-albumin conjugates, previously used for coupling the targeting peptide to nanogold (Curnis et al., 2013). The same approach can be exploited, in principle, for the preparation of other nanodrugs with different peptide ligands and different effector cytokines (e.g., IL12). This approach, besides reducing drug complexity, heterogeneity, and potential immunogenicity, may also reduce production costs, thereby facilitating nanodrug characterization and development.

## Data Availability

The original contributions presented in the study are included in the article/Supplementary Material, further inquiries can be directed to the corresponding authors.
