# Supplementary material for: Nanogold Functionalized With Lipoamide-isoDGR: A Simple, Robust and Versatile Nanosystem for αvβ3-Integrin Targeting
Source: Front Chem. 2021 May 28;9:690357. doi: 10.3389/fchem.2021.690357 (PMC8194705; doi:10.3389/fchem.2021.690357)
Supplement: Supplementary file 1 [file DataSheet1.docx]

Supplementary Material

# Supplementary Methods

**Optimization studies for the preparation of TNF-loaded gold nanoparticles**

To optimize the method for efficient adsorption of TNF to nanogold surface, we prepared aliquots of gold solution (1 ml) with a final pH adjusted to pH 5.5, 6.2, or 7.0 and 8.0 with sodium hydroxide. These aliquots were then mixed with various amounts of TNF (range 0-320 µg in 100 µl) in 5 mM sodium citrate buffer, pH 6.0, for the conjugation at pH 5.5 and 6.2, or in 5 mM sodium phosphate buffer, pH 7.0, for the conjugation at pH 7.0 and 8.0. After 15 min of incubation, an aliquot (100 µl) was removed, mixed with an equal volume of 10% (w/v) sodium chloride, and analyzed by UV-visible spectroscopy.

**Optimization studies for the preparation of bifunctional gold nanoparticles bearing compound 1 and TNF (1-Au/TNF)**

To prepare nanogold functionalized different amounts of TNF and compound **1**, we exploited a one-step conjugation procedure. To this aim, we prepared pre-mixed solutions containing a fixed amount of TNF (range 80–160 µg) and various amounts of compound **1** (range 5–320 µg) in 100 µl of 5 mM sodium phosphate buffer, pH 7.4. Each mixture was then added to 1 ml aliquots of nanogold, with pH adjusted to ~7.5 with sodium hydroxide, and left to incubate for 30 min at room temperature. The products were mixed with 100 µl of 0.5% HSA in water (in 25 µl aliquots every 2 min, for 4 times) and left to incubate for 10 min at room temperature. The products were then centrifuged at 13,000xg for 15 min. The pellets were resuspended in 5 mM sodium phosphate buffer, pH 7.33, containing 0.05% HSA (*storage buffer*). The centrifugation/washing steps were repeated twice. The final product was resuspended with 1 ml of storage buffer and stored at 4 °C. Optimization studies for preparing control gold nanoparticles functionalized with TNF and compound **2 or 3** (instead of compound **1**) were prepared as described above, using a fixed amount of TNF (160 µg).

# Supplementary Results

## Determination of the optimal pH for the preparation of TNF loaded nanogold

It is well known that the adsorption of proteins to sodium citrate-stabilized gold nanoparticles depends on the pH of the colloidal gold and protein concentration (Thobhani et al., 2010). Thus, to optimize the method for coupling TNF to nanogold, we investigated the effect of various amounts of TNF added to 1 ml aliquots of colloidal gold with pH adjusted to 5.5, 6.2, 7.0, and 8.0 (range 0–32 µg/ml TNF, final concentration). The adsorption of TNF to nanogold was then checked using the salt-induced aggregation assay. The results showed that the optimal pH for adsorption of TNF to colloidal gold was between 7.0–8.0, with TNF concentration >8 µg/ml (**Fig. S2**). At lower pH values, the addition of TNF caused particle agglomeration, even before the addition of sodium chloride. Based on these findings, the following conditions were selected for further studies: 16 µg/ml of TNF (final concentration) in 5 mM sodium phosphate buffer, pH 7.4, added to 1 ml of nanogold, pH 7.4–7.5.

## Determination of the optimal amount of TNF and compound 1 for the preparation of a bifunctional gold nanodrug

To verify the presence of compound **1** and TNF on nanoparticles, we measured the capability of nanodrugs to form molecular sandwiches using the anti-PEG mAb/anti-TNF pAb sandwich assay.

In parallel, we tested also control nanodrugs prepared with TNF and compound **2**. Both nanodrugs showed bell-shaped dose-response curves suggesting that the preparation of bifunctional nanoparticles is feasible when optimal doses of PEG-ligands and TNF are used (**Fig. S3A and S4**). In particular, maximal binding was obtained with nanodrugs prepared with 16 µg of TNF and 2-4 µg of ligands per ml of nanogold. Of note, no binding was observed when the assays were carried out using microtiter plates lacking the anti-PEG mAb *(data not shown*), suggesting that the binding was specific.

To verify the presence of functional compound **1** on nanoparticles, we then analyzed the binding of conjugates using the αvβ3/anti-TNF pAb sandwich assay. The results showed that nanogolds prepared with compound **1** and TNF, but not those prepared with compound **2** and TNF, could bind αvβ3 (**Fig. S3B and not shown**), pointing to the presence of functional *iso1*. Accordingly, the maximum binding was achieved when nanoparticles were prepared using 12-16 µg of TNF and 2–4 µg of compound **1** (**Fig. S3B).** Based on these findings, the following conditions were selected for the preparation of a large amount of nanogold functionalized with TNF and compound **1** or **2**: 100 µl of a pre-mixed solution containing 160 µg of TNF and 30 µg of compound **1** (or 20 µg of compound **2**) in 5 mM sodium phosphate buffer, pH 7.4, added to 1 ml of nanogold, pH 7.4–7.5.

# Supplementary References

Thobhani, S., Attree, S., Boyd, R., Kumarswami, N., Noble, J., Szymanski, M., and Porter, R.A. (2010). Bioconjugation and characterisation of gold colloid-labelled proteins. *J Immunol Methods* 356**,** 60-69.

**Supplementary Tables and Figures**

**Table S1**: Molecular mass of compound **1**, **2**, **3** and **4**, as determined by electrospray ionization mass spectrometry analysis (ESI-MS).

| **Conjugate** | **Compound code** | **Deconvoluted monoisotopic mass** | |
| --- | --- | --- | --- |
|  |  | Expected (Da) | Found (Da) |
| *iso1*-PEG_11_-LPA | **1** | 1428.61 | 1428.62 |
| Cys-PEG_11_-LPA | **2** | 1004.45 | 1004.44 |
| *iso1*-PEG_3_-LPA | **3** | 1104.44 | 1104.44 |
| Cys-PEG_3_-LPA | **4** | 680.27 | 680.26 |

**

**

**Figure S1. Mass spectra of compound 1 (A), 2 (B), 3 (C) and 4 (D).**

**
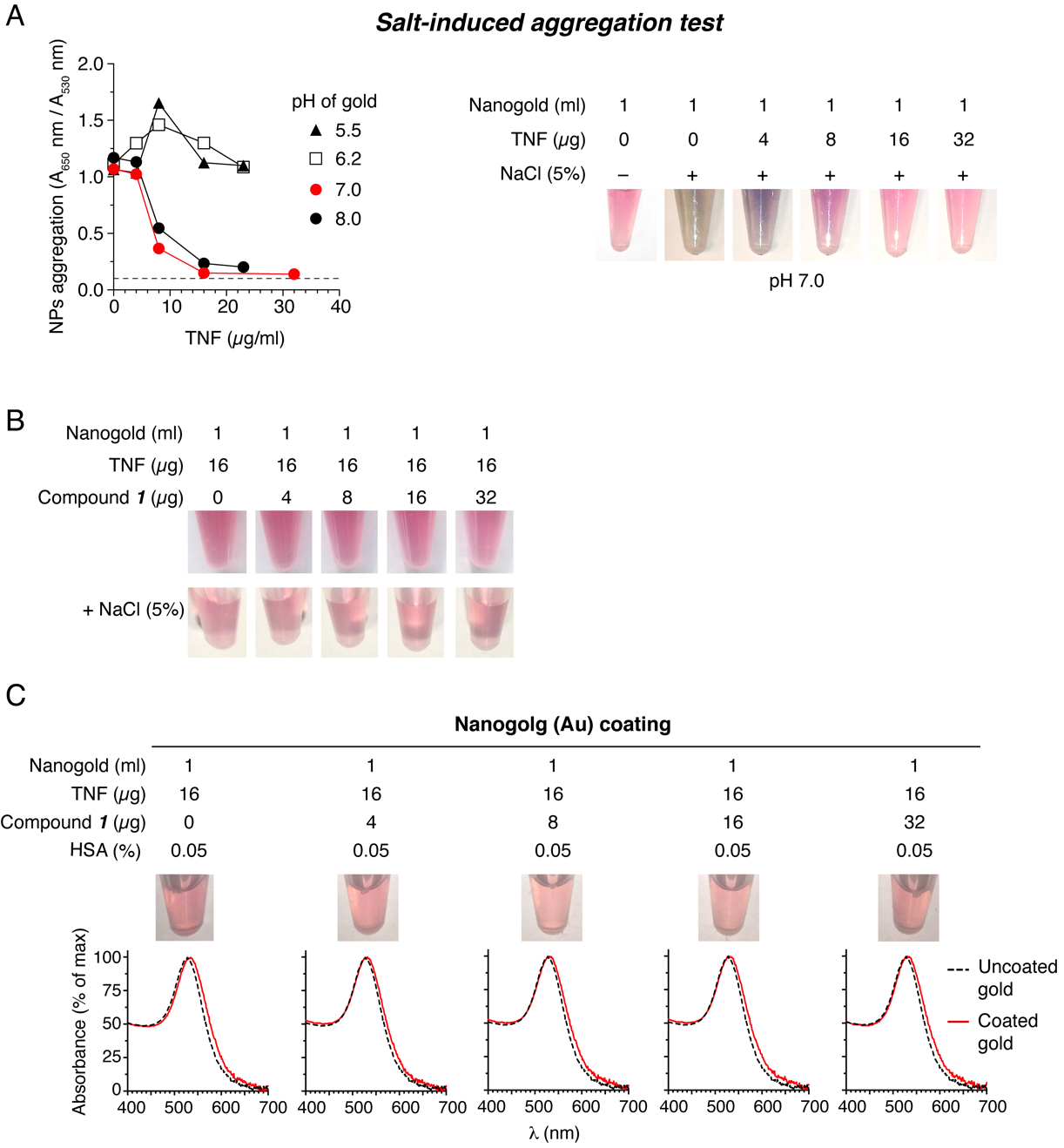
Figure S2. Optimization studies for the preparation of nanogold bearing TNF and compound 1.**

(**A**) Determination of optimal pH for TNF adsorption to colloidal gold nanoparticles (*see also Supplementary Methods*). An increase of A_650_/_530_ nm ratio and a marked change of color indicate nanoparticle aggregation. Note that nanogold coating with TNF >8 µg/ml at pH 7.0–8.0 prevents nanogold aggregation. Thus, pH 7.5 was selected.

(**B**) Effect of compound **1** and TNF on nanogold aggregation at pH 7.5.

One hundred microliters of pre-mixed solutions containing a fixed amount of TNF (160 µg/ml) and various amounts of compound **1** (range 0–320 µg/ml) were added to one ml aliquots of nanogold at pH ~7.5. After incubation, aliquots of the product were removed and then subjected to salt-induced aggregation (+ NaCl). Note that none of the mixtures caused nanoparticle aggregation.

(**C**) Effect of HSA on nanogold functionalized with compound **1** and TNF.

Nanoparticles described in **B (*upper panel****)* were mixed with 100 µl of 0.5% HSA (final concentration 0.05%), and the resulting products were analyzed by spectrophotometry.

Note that HSA can be added as a second stabilizer without causing nanoparticle aggregation.


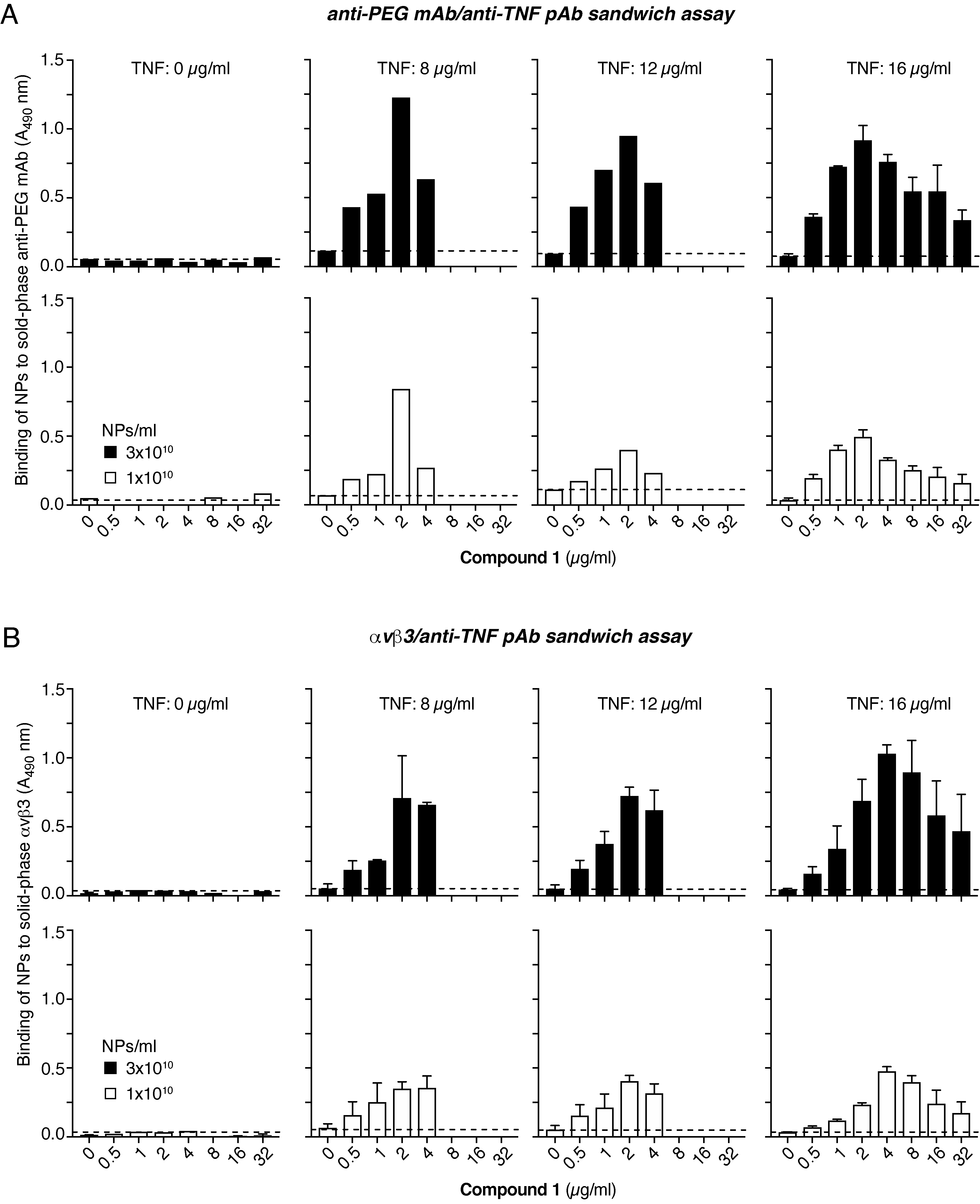


**Figure S3.** **Optimization studies for the preparation of bifunctional NPs bearing TNF and compound 1.**

Binding of NPs to anti-PEG mAb (**A**) or to αvβ3 (**B**) adsorbed onto microtiter plates (*solid-phase*), as detected with anti-TNF pAb (*anti-PEG mAb/anti-TNF pAb and αvβ3/anti-TNF pAb sandwich assay*)*. Bars*, mean±SE of 1-3 independent preparations.


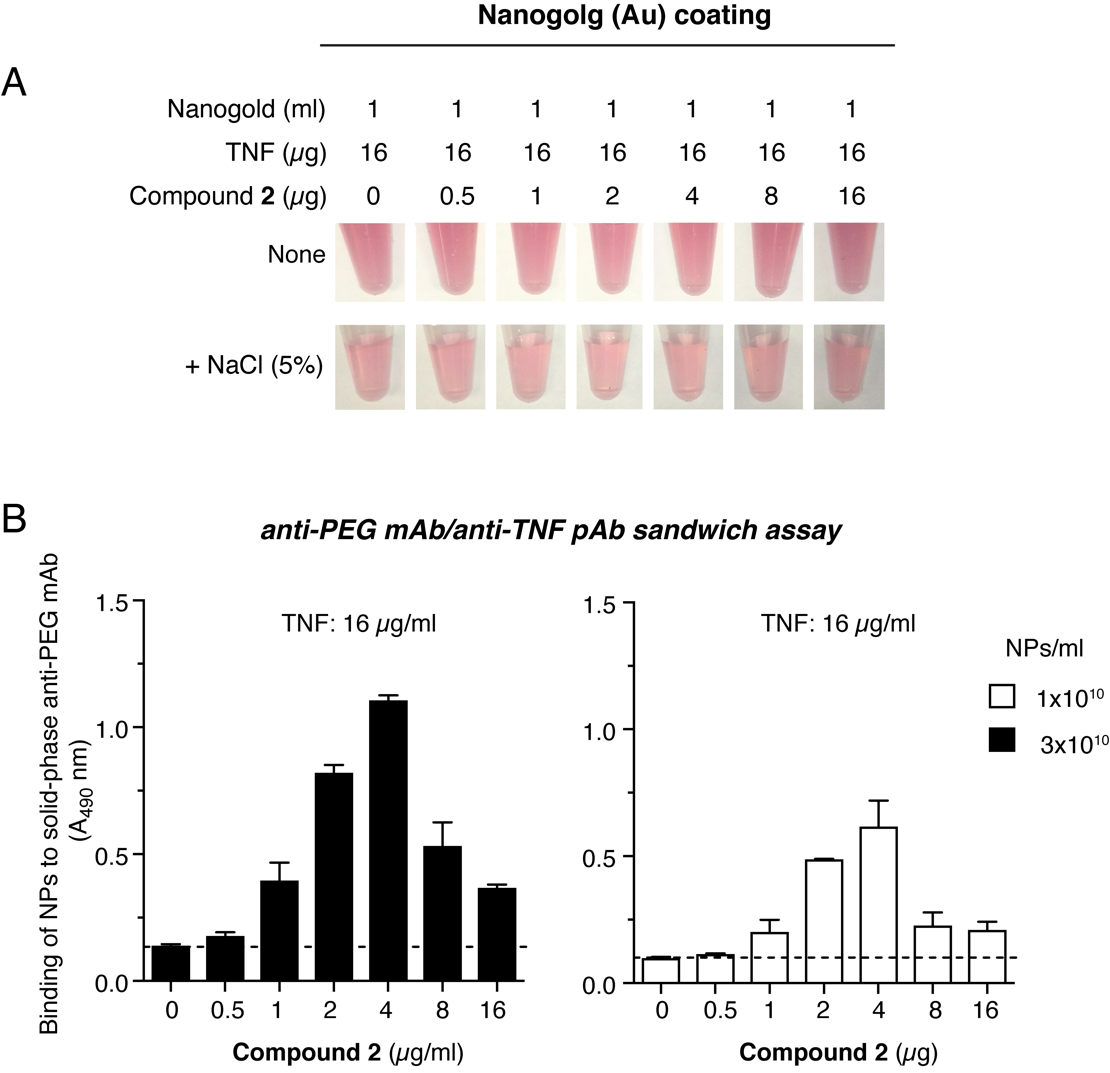


**Figure S4.** **Optimization studies for the preparation control NPs bearing TNF and compound 2.**

(**A**) *Effect of compound* ***2*** *and TNF on nanogold aggregation (see methods).*

Note that none of the mixtures caused nanoparticle aggregation.

(**B**) Binding of nanodrugs described in **A (*upper panel***) to an anti-PEG mAb adsorbed onto microtiter plates (*solid-phase*), as detected with an anti-TNF pAb (*anti-PEG mAb/anti-TNF pAb sandwich assay*)*. Bars*, mean±SE of duplicates.


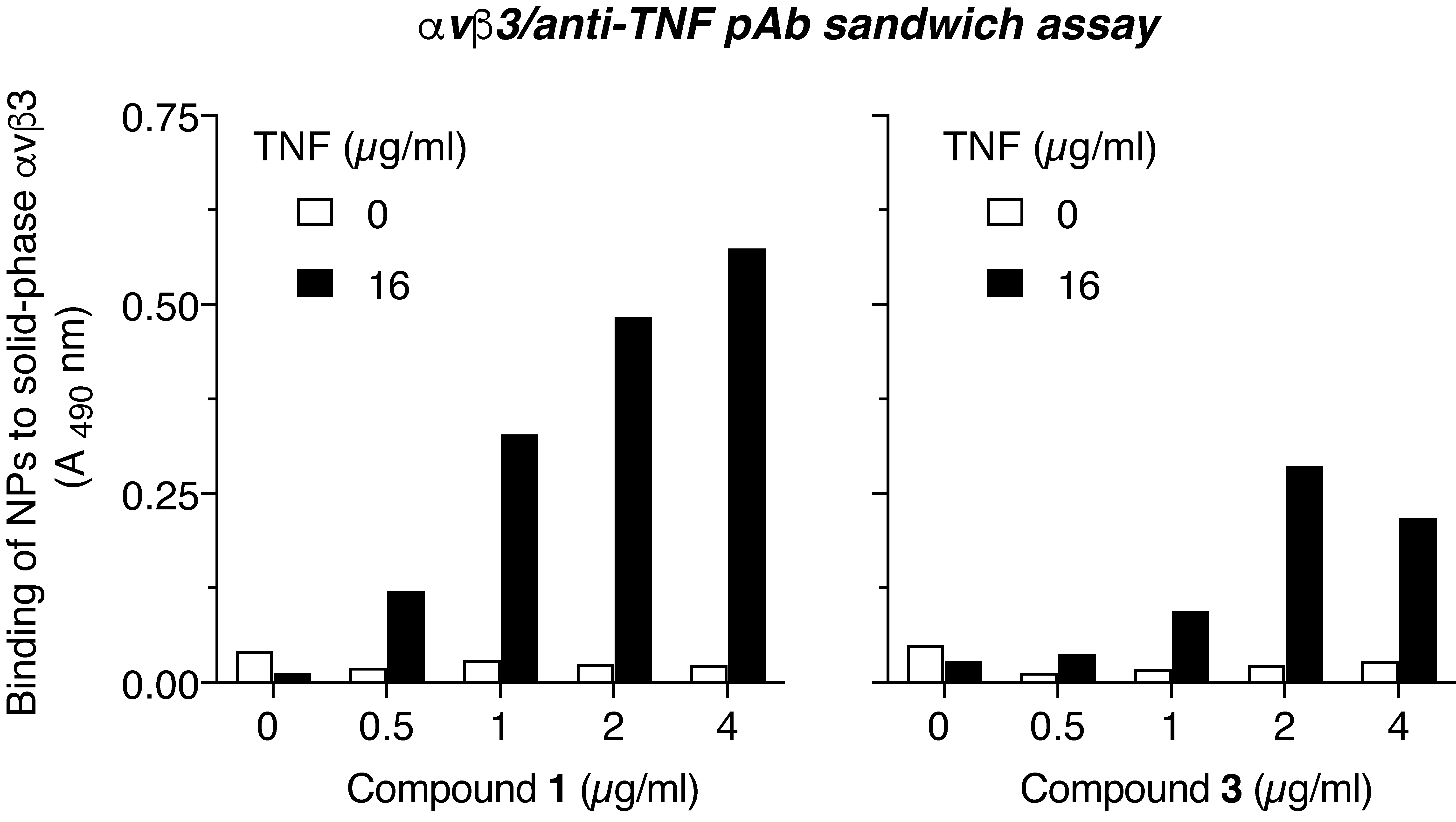


**Figure S5. NPs bearing compound 1 and TNF bind αvβ3 better than NPs bearing compound 3 and TNF.**

Gold nanoparticles functionalized with or without TNF and various amounts of compound **1** or **3** were tested (3x10^10^ NPs/ml) for their capability to bind αvβ3-coated plates and detected with anti-TNF pAb (*αvβ3/anti-TNF pAb sandwich assay)*.


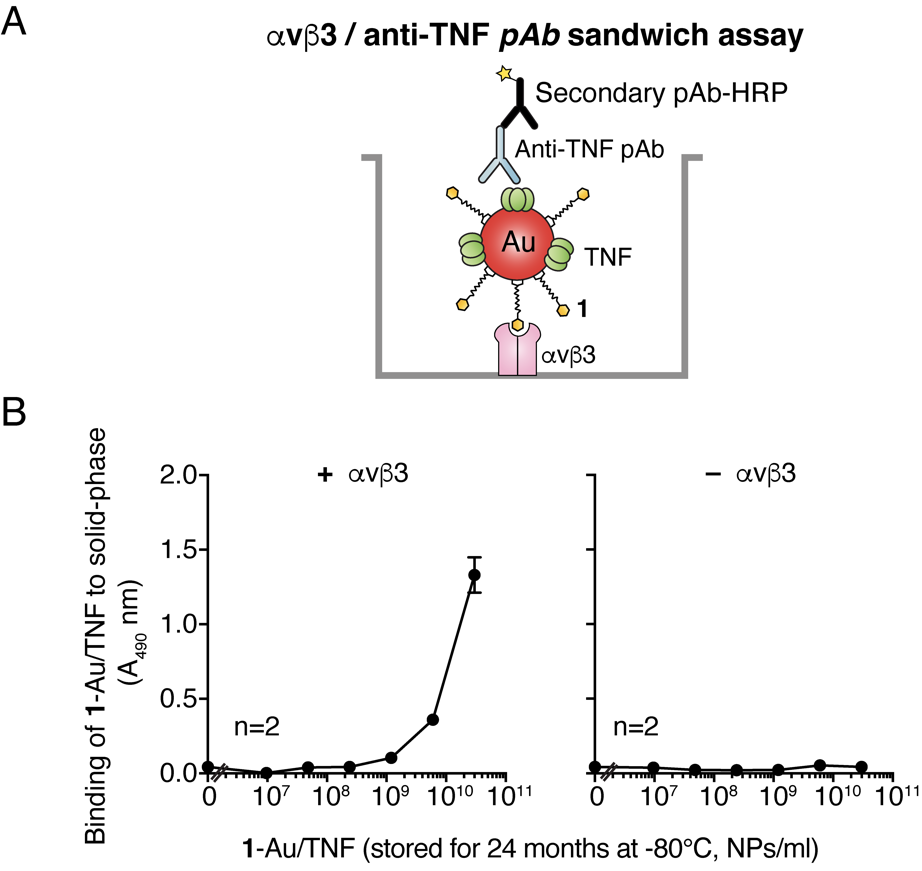


**Figure S6. Binding of 1-Au/TNF to microtiter plates coated with or without αvβ3 after storage for 24 months at −80 °C.**

A) Schematic representation of the assay.

B) Binding curves of **1**-Au/TNF. Mean ±SE of duplicates.

**
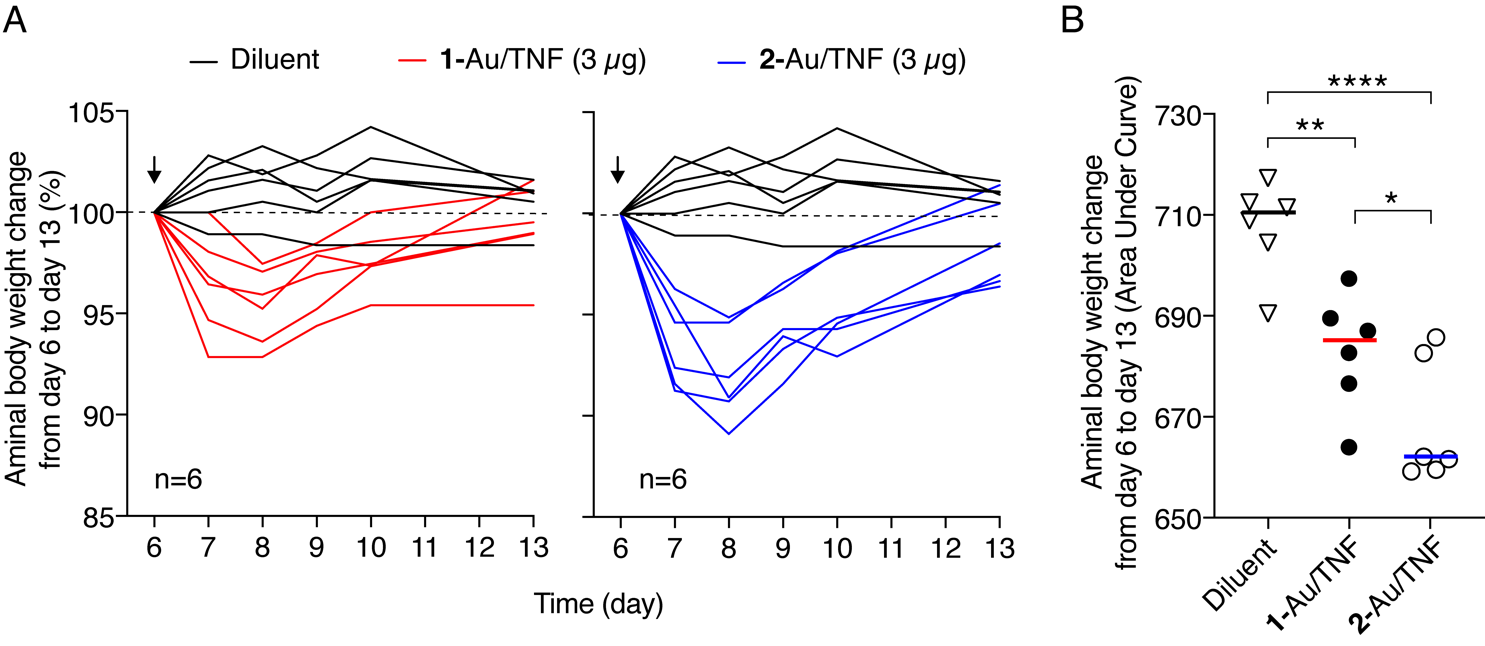
**

**Figure S7. Effect of 1-Au/TNF or 2-Au/TNF on the body weight of WEHI-164 tumor-bearing mice.** Tumor-bearing mice were treated at day 6 after tumor implantation with a dose of **1**-Au/TNF or **2**-Au/TNF equivalent to 3 µg of biologically active TNF (i.v. in 0.9% sodium chloride). Control mice were treated i.v. with 0.9% sodium chloride (Diluent).

(**A**) Change of body weight in each mouse from day 6 to day 13 after treatment (*arrow*).

(**B**) Area Under Curve (AUC) values of each mouse as reported in panel (**A**). The AUC was calculated using the GraphPad Prism software. *, P < 0.05, **, P < 0.01, ****, P < 0.0001 by one-way ANOVA with post-hoc *Fisher's least significant difference test*.
